# PerLE: An “Open Source”, ELearning Moodle-Based, Platform. A Study of University Undergraduates’ Acceptance

**DOI:** 10.3390/bs8070063

**Published:** 2018-07-16

**Authors:** Rocco Servidio, Michael Cronin

**Affiliations:** Department of Languages and Educational Sciences, University of Calabria, Via Pietro Bucci, Cube 20/B, 87036 Arcavacata di Rende, Italy; cronin@unical.it

**Keywords:** eLearning, blended learning, technology acceptance model, technology acceptance model extensions

## Abstract

The implementation of innovative eLearning platforms offers numerous benefits, but it is important to understand individual acceptance and use of new technological systems in the educational setting. This study adopts a modified version of the Technology Acceptance Model (TAM), including three service quality constructs as external variables, to assess students’ acceptance of PerLE, a Moodle-based eLearning platform developed at the University of Calabria (Italy). A six-section questionnaire, which was based on previous studies, was administered to 293 undergraduate students. Results show that the quality of online courses is the main construct that affects students’ acceptance of PerLE. We found also that the PerLE user interface was a critical issue, requiring improvements to facilitate ease of use. In addition, the study underlines the important influence of Technical Support as an antecedent to the two main constructs of the TAM: PerLE Usefulness and PerLE Ease of Use.

## 1. Introduction

When used in educational contexts, eLearning environments have numerous aspects that require constant evaluation [1]. Evaluation is essential to manage the processes which are involved in the use of technological tools to learn, discover, collaborate, and share knowledge [2,3]. We need to understand students’ motivation, experiential perception, and disposition to use digital learning materials. We also need to prepare didactic activities in a virtual environment that students find comfortable and consider useful.

Blended learning which focuses on learner-centeredness is a significant component of current university educational approaches [4,5]. Furthermore, there has been a greater focus on the integration of digital tools to promote the use of social media applications as well as information and communication technologies to foster interesting and play-oriented learning [6,7]. From an instructional design point of view, three principal considerations come into play in which the concern is to guarantee an educational environment responsive to user needs and abilities [8]. First, it is essential that users perceive the proposal as useful; therefore, learning paths, objectives, and outcomes need to be clearly communicated to users. Second, user disposition to engage with eLearning course contents in a digital environment is enhanced by the promotion of a sense of “community”, bringing shared collaboration to defined learning pathways. Third, the acquisition of digital skills and competence has been stipulated by the European Commission in its promotion of the European Framework for Digitally Competent Education Organizations.

Although formal instruction is still predominant in the classroom, the diffusion of web-based and related technologies extends the range of application of instructional design practices with the support of numerous software solutions. These both facilitate the learning process and motivate students to use them autonomously [9].

The current educational trend is to design not only more advanced eLearning environments but also to apply consolidated theoretical models to evaluate both the levels of users’ interaction and their acceptance of the system [5]. A recent study cites several eLearning scholars who maintain that few universities fully exploit the potential benefits of eLearning; the study argues that existing research has concentrated too much on staff perspectives. Instead, the paper argues that an understanding of student acceptance of eLearning is crucial to the design, implementation, and management of a productive eLearning environment [10].

We resolved to adapt the Technology Acceptance Model (TAM), which was originally proposed by Davis [11], who suggested that ease of use and usefulness of a technological system affects the user’s intention to use it. Specifically, we used the TAM as the conceptual and methodological framework to assess those factors that may affect student acceptance to use PerLe, which has not yet been evaluated in these terms. Thus, our analysis focuses on the following aims:

- To investigate the acceptance factors for the PerLE platform for students enrolled on blended learning courses;
- To examine the profile of students involved in blended learning teaching modalities;
- To evaluate the importance of the role of technical support, user interface, and course materials in influencing user response to the platform.

The paper has seven sections: the present introduction, the theoretical background for the study, the research hypotheses, method, results, discussion, and the conclusion.

## 2. The Technological Acceptance Model in eLearning

The TAM constitutes a valid approach in explaining and predicting both acceptance and usage behaviour of information technologies [12]. It maintains that the success of a technological system can be determined by user acceptance, and that acceptance is measured by two factors: perceived usefulness (PU) and perceived ease of use (PEOU). These factors are the primary predictors of an individual’s behavioural intention (BI) to use technological tools. For Davis [11], PU means that users trust that a particular system will increase their performance, while PEOU refers to the belief that using a particular system will result in a decrease in the effort necessary to achieve a given objective.

Increasingly, international researchers in education have adopted the TAM to explore the extent of learner acceptance of eLearning systems as new learning environments [1,5,13,14,15]. Findings report a prevailing positive learner attitude towards eLearning systems. Recently, empirical evidence has provided valuable rational explanations which confirm that it is appropriate to extend the original version of TAM by including external variables (e.g., technical support, social norms, computer self-efficacy) as determinants of the usefulness construct [5].

One study applied the TAM to evaluate methodological and technological improvements deriving from the introduction of a Moodle-based eLearning system in an online Italian university. It considered all system users and made use of additional information related to the system in the evaluation. Results confirmed the validity of extending the component criteria of the TAM to embrace other factors identifiable as defining characteristics of a given system [16]. In this context, an analytical use of TAM facilitates testing of eLearning systems to verify their didactic efficacy as well as to consider the emerging tools and functions which enable a wide range of instructional design uses, further enabling student response and intervention. The following section offers a description of PerLE, its characteristics and functions.

### The Description of the PerLE Platform

**Figure 1:**
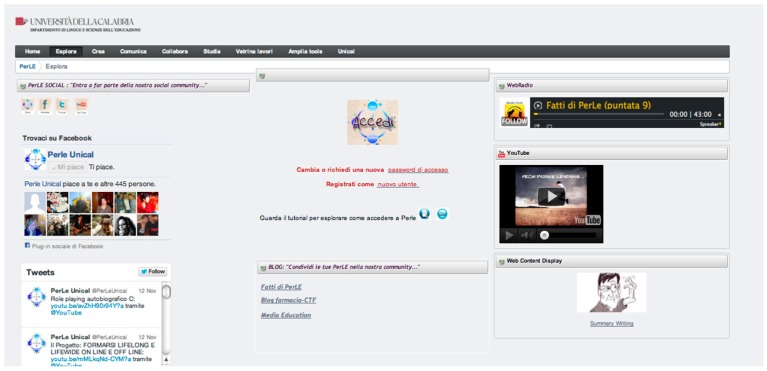
The PerLE homepage.

PerLE is an open-source digital learning platform and environment whose architecture offers students the opportunity to develop digital skills, to collaborate on community-based activities, and to display their work as well as personal and group initiatives. It also provides the institutional function of housing courses, materials, and project activities, making use of the different approaches available in Blended Learning models. Figure 1 illustrates how the platform is presented to the user, offering access to areas of social media-supported community activities, courses, blogs, and streaming channels. 

**Figure 2:**
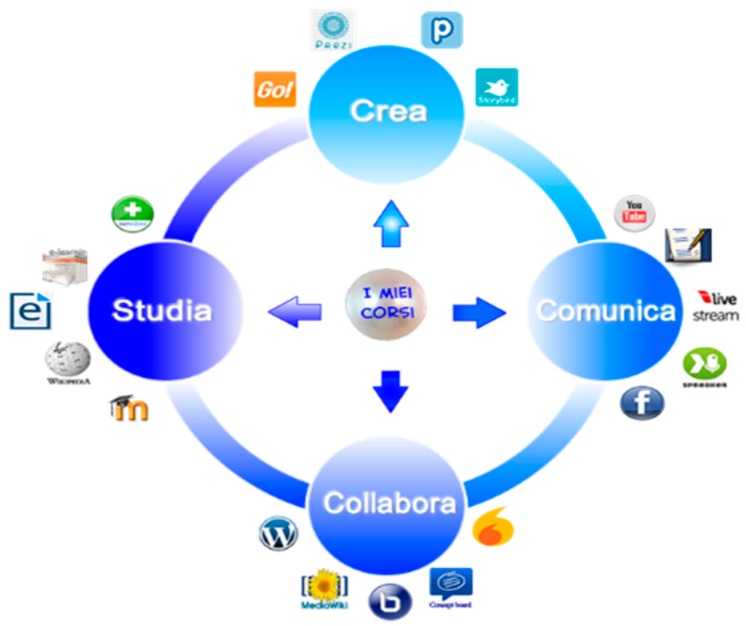
PerLE icon with access to materials, courses, and external software.

The formal study component is Moodle-based and employs a modularity-based approach, which is understood as the freedom to integrate useful learning objects, software tools, and media, favouring responsiveness to user needs. The interactive icon shown in Figure 2 offers access to web tools to support learning (Create), promotes communication and exchange (Communicate), makes tools available to eliminate distances (Collaborate), and provides access both to institutional courses and open learning sources.

This enables experimentation with blended learning initiatives aimed at stimulating students to develop digital skills and competencies [17,18]. The initiative was a response to developments in eLearning and comprised an opportunity to promote learning in a scenario characterized by principles of community of practice as described by Wenger [19], in a residential campus university with approximately 30,000 students. The goals in developing PerLE included:

- Encourage self-directed activity management.
- Promote collaborative learning/community of practice.
- Develop digital competence and literacy.
- Integrate use of tools and social media.

In the PerLE platform, didactic materials include:

- Provision of support materials for study on particular degree courses on the rotation model;
- Projects for the completion of activities using presentation formats familiar in MOOCs (flipped classroom approach);
- Projects managed according to a lab rotation model with class/lab time monitoring and support of student activity in the digital environment.

The designers of PerLE believe that the fostering of learning collaboration in an environment open to a range of integrated components is sustainable if the digital environment satisfies expectations related to the use and acceptance of the eLearning proposal. Therefore, access to the platform, navigation to specific course content, and use of functions and tools must be efficient and responsive. These factors promote a positive perception of platform use as well as the provided service, which responds to needs ranging from paced performance on coursework to varied forms of communication, exploration, sharing, and collaboration. To facilitate ease of access to options and menus, PerLE offers support for problems of access, accreditation, and general platform use through tutor support and a help service. Our concept of Blended Learning in PerLE is a response to user requirements and is to be adapted and varied according to perceived needs. This involves gauging the acceptance, motivation, ease of use and perception of efficacy of a potential user, a process that is concomitant with other forms of evaluation carried out by course managers.

## 3. The Study: Research Design and Hypotheses

Results from recent research highlight the TAM as one of the most important information systems theories in eLearning acceptance [16,20,21]. Taking into account established research [1,22,23], we extend the TAM model by exploring which external variables affect learners’ intention to use PerLE for eLearning. According to Sánchez and Hueros [22], extrinsic and intrinsic factors can indirectly influence the acceptance of technologies via both perceived usefulness and perceived ease of use. Thus, we selected external variables relative to their relevance to the educational context and for their effects on the TAM constructs. Within the TAM framework, the proposed research model includes, as external variables, three service quality constructs.

The first construct, Technical Support (TS), consists of intervention by technical staff to assist students in using the platform effectively; it also includes support functions such as telephone, internal messaging, email, and personal tutoring. Quality of technical support influences students’ interest in the acceptance of the technology because as it is related to overall satisfaction [22]. A recent meta-analysis showed how researchers usually extend the basic TAM research model by identifying some prior factors, including technical support [24]. We thus propose the following hypotheses:

**H1.**  Technical Support has a positive effect on PerLE Usefulness.

**H2.**  Technical Support has a positive effect on PerLE Ease of Use.

The second construct, PerLE User Interface (PUI), is defined as the extent to which students find the system interface easy to understand by promoting and facilitating the interaction between students and the educational tool. The quality of the User Interface is critical to the design and development of technological applications and it should satisfy the usability criteria [25,26]. Thus, we identified the hypotheses:

**H3.**  PerLE User Interface will positively affect PerLE Usefulness.

**H4.**  PerLE User Interface will positively affect PerLE Ease of Use.

Lastly, the third construct, Online Course Lesson (OCL), defines the extent to which educational learning contents are developed to fit students’ needs. This function denotes all educational materials that the teachers create and publish in their own dedicated course areas. It is a repository where students can download or initiate interactive didactic contents on specific topics. Currently, there are several types of course content available, ranging from course-specific materials to guided projects on specific subjects.

The quality of online educational materials is another central factor in the success of an eLearning platform [1]. Students are the focus of the didactic activity and their perceptions regarding ease of use and usefulness of online lessons have important implications for supporting their motivation to learn as well as their improvement in practical skills [13,27]. This leads to the following hypotheses:

**H5.**  Online Course Lesson has a positive effect on PerLE Usefulness.

**H6.**  Online Course Lesson has a positive effect on PerLE Ease of Use.

In accordance with the TAM model, two belief variables, PerLE usefulness and PerLE ease of use, were included and represent the determinant variables that influence user acceptance of the technological system. Perceived usefulness of PerLE can be defined as the “degree to which an individual believes that using a particular system will enhance his or her performance” [11]. For its part, perceived PerLE Ease of Use represents “the degree to which a person believes that using a particular system would be free of effort” [11]. As suggested by Davis [11], perceived ease of use directly affects perceived usefulness. Other studies have confirmed this relationship [22,28]. Therefore, we propose the following hypotheses:

**H7.**  PerLE Ease of use affects PerLE Usefulness.

**H8.**  PerLE usefulness has a positive effect on PerLE System Usage.

**H9.**  PerLE Ease of Use has a positive effect on PerLE System Usage.

The dependent variable is PerLE System Usage (PSU), which defines students’ use of the system and their behaviour. We excluded the Intention to Use variable because, as previous studies indicate, even if a student’s attitude towards web-based learning is positive, they may have no intention to use one unless their lecturers consider this necessary [22,29].

## 4. Materials and Methods

The following sections provide a detailed description of how the study was conducted in terms of participants, data collection, instrument design; it also includes the procedure for statistical analysis.

### 4.1. Participants and Data Collection

Our study involved 293 undergraduates who used PerLE during course-related activities. Only students who had accounts and had used PerLE for nearly a year prior to the study completed the questionnaire. These parameters were set to test the effective learning potentialities of PerLE in terms of acceptance. An email was sent to all second-year students (registered with the system via their institutional email addresses) on all courses available in PerLE, inviting them to participate. The response rate within the specified time was 36.6%. Table 1 shows the characteristics of the sample explored with three questions.

The data were collected from November 2016 to February 2017 using the open source software, LimeSurvey. The language used was Italian. The first page of the questionnaire emphasized the voluntary nature of participation and the possibility to interrupt questionnaire completion at any time. Anonymity was guaranteed and participants were informed that the data would be used only for research. Other information collected included self-perception of personal competences in the use of information technologies, with 63.6% of the respondents reporting a basic level, 73.7% intermediate, and 54.6% advanced, with time spent using PerLe per week (M = 5.10, SD = 2.10). The study procedures were in compliance with the Helsinki Declaration of 1975 and the Code of Ethics of the Italian Psychology Association (AIP).

### 4.2. Design of Instruments

During the development of the questionnaire, the items of the TAM constructs were adapted, with minor language modifications, from previously validated questionnaires used in the context of eLearning frameworks (A1, Appendix A). The questionnaire sections were structured as follows: the first part collected information on Technical Support, the second part assessed the PerLE User Interface, and the third section assessed the Online Course Lesson. In the fourth section, students were invited to assess PerLE Usefulness, assessing PerLE Ease of Use in the fifth section and PerLE System Usage in the sixth section. All the items were rated using a seven-point Likert scale from 1 (strongly disagree) to 7 (strongly agree), where 4 was a neutral answer, according to the respective original scales.

The last part of the questionnaire included items with different categories of answers, which related to the demographic profile of the students and other relevant information about eLearning and the use of PerLE.

### 4.3. Data Analysis Procedure

The Statistical Package for the Social Sciences (SPSS ver. 24) was used to perform descriptive, reliability, and other statistical analyses. Participants who did not respond to one or more items, with the exception of the profile section, were excluded. Convergent and discriminant validity were computed to assess the construct reliability. Convergent validity tests whether the items of a construct that are expected to be related are highly correlated [30]. Composite Reliability (CR) with values greater than 0.70 and Average Variance Extracted (AVE) greater than 0.50 are indicators for convergent validity [12]. Table S1 shows the estimates, standard error, R-square, Cronbach’s alpha, CR, and AVE of the current model. All the measures exceeded the minimum values. Values for the Cronbach alpha test were above 0.81, showing good internal consistence reliability [30]; the CR ranging from 0.82 to 0.95 and the AVE from 0.53 to 0.76, demonstrating good convergent validity. Discriminant validity, for its part, tests whether the measure is a reproduction of other variables. Discriminant validity can be demonstrated if the square root of the AVE for a construct is greater than the relationship between the construct and all other constructs in a measurement model [12]. The discriminant values are shown in Table 2, with results showing good properties of the construct.

A Structural Equation Modeling (SEM) was produced to test the proposed TAM aimed at examining the acceptance of PerLE. Data were analyzed by using Mplus (ver. 6.12; [29]). Taking into account the ordinal nature of the study variables, we adopted a maximum likelihood (MLM) estimator with standard errors and a mean-adjusted chi-square test statistic that is robust to non-normality [31]. The MLM estimator provides the Satorra-Bentler scaled chi-square (S-B χ^2^) [32], an adjusted and robust measure of fit for non-normal data which is more accurate than the ordinary chi-square test [33,34]. To test model accuracy, the study applied the most commonly used fit indices and related cut-off values. Specifically, the ratio of chi-square is relative to its degrees of freedom (S-B χ^2^/*df*), where values of less than 3.0 were considered to reflect a good model fit [30]. Results for this test included: Comparative Fit Index (CFI) with recommended values of >0.95; the Tucker-Lewis Index (TLI) with values above 0.95 [35]; the Root Mean Square Error of Approximation (RMSEA) with values of cut-off <0.05; and the Standardized Root Mean Square Residual (SRMR) <0.05 [36].

Potential model adjustments were based on modification indices, as provided in the Mplus output. Following the advice of Byrne [36], we considered only correlation errors within each group of variables whereas cross-loadings correlations were excluded.

## 5. Results

The results of the CFA showed a good measurement fit to the data (Table 3). The proposed model indicated that all items were reliable indicators of the hypothesized constructs they were supposed to measure.

**Figure 3:**
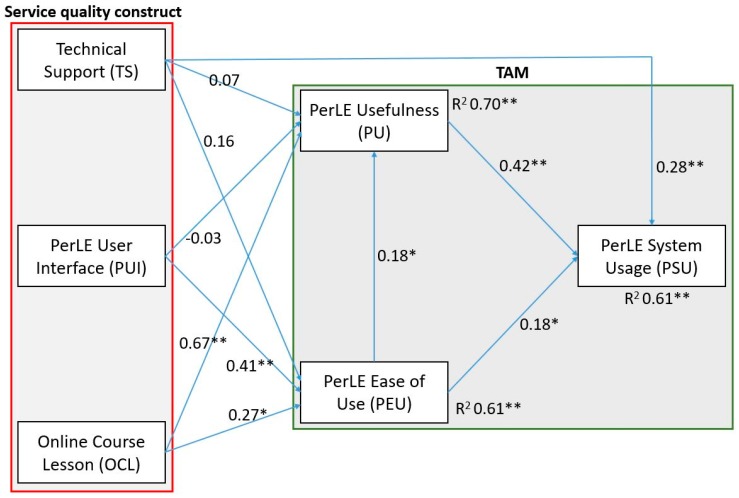
Standardized results of the model. * *p* < 0.05; ** *p* < 0.001. R^2^ (R-Square) shows the percentages of variance explained by the predictor variables. The red line indicates the variables of the service quality constructs while the green line indicates the conventional TAM variables.

Figure 3 shows the causal relationship between the constructs and the standardized path coefficients.

We found no significant relationship between Technical Support and PerLE Usefulness (β = 0.07, *p* > 0.05), rejecting both hypothesis 1 and PerLE Ease of Use (β = 0.16, *p* > 0.05) as well as hypothesis 2.

Hypothesis 3, which was rejected, explored the relationship between PerLE User Interface and PerLE Usefulness (β = −0.03, *p* > 0.05). Hypothesis 4, which involved PerLE User Interface and PerLE Ease of Use (β = 0.41, *p* < 0.001), was accepted. Hypothesis 5 supported the relationship between Online Course Lessons and PerLE Usefulness (β = 0.67, *p* < 0.001). Online Course Lesson was also positively related to PerLE Ease of Use (β = 0.27, *p* < 0.05), confirming hypothesis 6. A positive and significant relationship was found between PerLE Ease of Use and PerLE Usefulness (β = 0.18, *p* < 0.05), confirming hypothesis 7. PerLE System Usage was related positively and significantly both to PerLE Usefulness (β = 0.42, *p* < 0.001), supporting hypothesis 8 and to PerLE Ease of Use (β = 0.18, *p* < 0.05), confirming hypothesis 9. Lastly, we found a positive and significant relationship between Technical Support and PerLE System Usage (β = 0.28, *p* < 0.001). The R-Square results indicate that the predictor variables (external variables) explained the 70% for PerLE Usefulness and the 61% for PerLE Ease of Use. In turn, PerLE Usefulness and PerLE Ease of Use explained the 61% for PerLE System Usage. The cause of the other remaining variance of the aforementioned predictors is unknown.

## 6. Discussion

This study explored the degree of student willingness to use the PerLE platform for blended learning in formal learning and as participants in an online community. Results corroborated the study model and supported most of the causal relationships defined in the proposed hypotheses.

Online Course Lesson is the most significant determinant directly affecting PerLE Usefulness. This result is consistent with previous studies, which underline the importance of producing stimulating and motivating online educational materials for students, providing them with external resources and services that meet their learning expectations [1,21,37]. The relevance of Online Course Lesson indicates that, when the level of educational contents increases, the students tend to regard the eLearning technology in a more positive manner. The current findings also illustrate that PerLE Usefulness is a key factor linking the exogenous variable Online Course Lesson to PerLE System Usage. This suggests that, when the students perceive the usefulness of a technological system, they are more motivated to use it to satisfy their personal expectations. This means that teachers need to create stimulating learning materials to encourage students to use them more extensively [5]. We interpret this result as an implicit recommendation to teachers to design their educational materials in a fashion that is responsive to this positive reaction and sensitive to the need for careful design. PerLE User Interface is another important determinant affecting PerLE Ease of Use, and the present result confirms the conclusions of previous studies [1,38]. We found that, when the students perceive the PerLE interface as easy to use because of its design quality, they wish to engage with PerLE because they consider it to be useful. However, the relationship between PerLE User Interface and PerLE Usefulness is not significant. This result could be an adverse effect arising due to approaches adopted in the design of the user interface with regard to the presentation of didactic content. Users may perceive the use of web applications external to the PerLE platform less positively than those internal to it, the latter offering greater opportunities for teacher monitoring and feedback. The Technical Support construct had an insignificant direct effect on PerLE Usefulness and PerLE Ease of Use. This outcome is partially consistent with a previous study, which found that Technical Support has a significant effect only with the construct Perceived Usefulness [22]. Nevertheless, our findings indicate that Technical Support is a significant antecedent to PerLE System Usage. This underlines the importance of Technical Support and the strategy should be to recruit staff trained to support teaching staff in creating appropriate educational content, in addition to supporting students in using the eLearning system. PerLE Ease of Use directly affects PerLE System Usage. The weak connection between these two TAM constructs should encourage platform managers to consider the opportunity of augmenting the system’s ease of use through good design and clear instructions to students. This result corroborates the importance of eLearning as a new educational approach and it is consistent with previous studies [13,21,39].

Our findings illustrate the key role of Usefulness with regard to the PerLE eLearning platform. Specifically, we found that Online Course Lesson influences this relationship and this suggests that the interviewees are prone to adopt the eLearning platform for learning.

### Limitations and Suggestions for Future Research

Our study presents a number of limitations to be addressed in future investigations. First, it tested the effects of three external variables on the two main TAM determinants (PerLE Usefulness and PerLE Ease of Use). On the basis of previous studies, we decided to select these because this research is the first attempt to evaluate the PerLE platform. We then investigated the students’ technological acceptance, gathering information on technical support, user interface, and course lessons. Future studies should consider the inclusion of other external variables to further extend the TAM, for example, computer self-efficacy [12], subjective norms [38], playfulness, etc. [4].

Second, this study focused only on university students and did not explore the perceptions of teachers on PerLE usage. Future research should address the perceptions of students and teachers and analyse the differences between them to identify their respective requirements. The number of teachers adopting eLearning is on the increase but not in adequate proportion to the numbers of students doing so.

Third, we have limited our evaluation to the assessment of the functionalities of the system; the nature of the results suggests the utility of investigating the implications of the positive and negative responses to engagement with applications that are both internal and external to PerLE.

Finally, the results of this study may be generalized to other research by considering the Moodle-based nature of the PerLE platform. On the other hand, as a result of the homogeneous nature of the sample, the current results cannot be generalized to other studies. Further investigations should be conducted to verify the validity of the present findings.

## 7. Conclusions

This study supports the importance of testing students’ acceptance of eLearning platforms, and, in particular, suggests that the quality of online courses is the main construct affecting student acceptance of the PerLE platform. Considering this result, it is essential to the success of an eLearning environment that priority be accorded to constructive course content design. Highly pertinent in this regard is an understanding of student expectations regarding educational materials, didactic resources and tools used for eLearning purposes.

The participants involved in the present study were satisfied by the current PerLe user interface. However, we found that participants disliked the Usefulness of the PerLe User Interface, indicating that it required some improvements, especially for enhancing the ease of interaction with the external educational software provided by the platform.

In addition, the weak relationship between PerLE Ease of Use and PerLE System Usage demonstrates that developers should ensure the interface be user friendly, supporting students in the course of their interactions. In general, the results of this study contribute to the literature by providing evidence on the students’ acceptance of PerLE, a Moodle-based platform in use for blended learning, by gathering information from undergraduates. The TAM constitutes a helpful theoretical approach to predicting and understanding student acceptance of use of PerLE. It also confirms that to motivate students to use an eLearning system, teachers should make learning more interesting for users with the support of PerLE; this can be achieved by integrating quality materials through stimulating use of the tools available via the platform. Further, teachers need support in designing more user-oriented eLearning content, to minimize difficulties for students in a productive use of the platform. The study underlines the influence of Technical Support as an antecedent of PerLE Usefulness and PerLE Ease of Use. The results of the study provide practitioners (teachers, technicians, and degree course managers) with insights on the use of PerLE in terms of design and the delivery of educational contents as well as on student interaction.

## Figures and Tables

**Table 1:** Demographic information on students participating in the survey.

	Frequency	Percentage
***Gender***		
Female	262	89.4
Males	29	9.9
Missing	2	0.7
***Age***		
18–22	252	86.0
23–27	30	10.2
28–32	7	2.4
33–47	2	0.7
Missing	2	0.7
***Degree course***		
Education	211	72.0
Law	64	21.8
Tourism	16	5.5
Missing	2	0.7

**Table 2:** Discriminant validity of the TAM-PerLE model.

	AVE	TS	PUI	OCL	PU	PEU	PSU
TS	0.53	**0.73**					
PUI	0.61	0.64	**0.78**				
OCL	0.60	0.60	0.68	**0.77**			
PU	0.76	0.56	0.63	0.75	**0.87**		
PEU	0.71	0.56	0.68	0.62	0.63	**0.84**	
PSU	0.70	0.57	0.60	0.60	0.67	0.58	**0.84**

Note. All the correlations are significant at *p* < 0.001. Values in diagonal (bold character) are the squared roots of the AVE.

**Table 3:** Goodness-of-fit statistics for the Tam-PerLE model.

Model	SBχ^2^ (*df*)	SBχ^2^/*df*	CFI	TLI	RMSEA 90% (CI)	SRMR
Tam-PerLE	474.44 (362)	1.31	0.97	0.97	0.03 (0.02, 0.04)	0.04

Note: SBχ^2^ = Satorra–Bentler scaled χ^2^; df = degrees of freedom; CFI = comparative fit index; TLI = Tucker-Lewis index; RMSEA = root mean square error of approximation; CI = confidence interval and SRMR = standardized root mean square residual.

## Supplementary Materials

The following are available online at http://www.mdpi.com/2076-328X/8/7/63/s1, Table S1: Reliability of the measured model.

## Appendix A

The full version of the questionnaire A1.
