# Supplementary material for: PerLE: An “Open Source”, ELearning Moodle-Based, Platform. A Study of University Undergraduates’ Acceptance
Source: Behav Sci (Basel). 2018 Jul 16;8(7):63. doi: 10.3390/bs8070063 (PMC6070931; doi:10.3390/bs8070063)
Supplement: Supplementary file 1 [file behavsci-08-00063-s001.zip › Questionnaire A1.pdf]

PerLE: an open source eLearning Moodle-based platform. A study of university undergraduates' acceptance

Appendix A. Questionnaire used in the study

A seven-point Likert scale was used to answer the questions of the proposed constructs:

1 = In completo disaccordo; 2 = Abbastanza in disaccordo; 3 = Poco in disaccordo; 4 = Neutrale; 5 = Poco d'accordo; 6 = Abbastanza d'accordo; 7 = Completamente d'accordo.

Item	Statement	Reference
Technical Support (TS)		
TS1	In caso di problemi tecnici su PerLE, si possono fare richieste al personale addetto durante l'orario di ricevimento	Items adapted from Sánchez and Hueros (2010)
TS2	In caso di problemi tecnici su PerLE, si possono inviare richieste via e-mail	
TS3	In caso di problemi tecnici su PerLE, si possono inviare richieste di aiuto tramite i servizi della piattaforma	
TS4	Gli amministratori dell'ambiente didattico PerLE offrono un buon supporto tecnico	
PerLE User Interface (PUI)		
PUI1	Il primo impatto con la "Home page" (pagina principale) dell'ambiente didattico PerLE è positivo	Items adapted from Liu et al. (2010)
PUI2	Sia il design che la grafica dell'ambiente didattico PerLE sono facili da comprendere e interpretare	
PUI3	Il menu dei comandi rispecchia bene la struttura dell'ambiente didattico PerLE	
PUI4	Il significato del testo e delle icone dell'ambiente didattico PerLE è chiaro	
PUI5	In generale, sono soddisfatto per il design dell'interfaccia dell'ambiente didattico PerLE	
PUI6	In generale, sono contento di interagire con l'interfaccia utente dell'ambiente didattico PerLE	
Online Course Lesson (OCL)		
OCL1	Il materiale di studio presente nell'ambiente didattico PerLE è interessante e stimolante	Items adapted from Sánchez and Hueros (2010)
OCL2	Il materiale di studio presente nell'ambiente didattico PerLE agevola il mio apprendimento	
OCL3	Il materiale di studio presente nell'ambiente didattico PerLE soddisfa le mie esigenze di studio	
OCL4	I materiali educativi presenti nell'ambiente didattico PerLE usano tecnologie innovative	
OCL5	In generale, sono soddisfatto del design e della qualità del materiale di studio presente nell'ambiente didattico PerLE	
PerLE Usefulness (PU)		
PUI1	L'ambiente didattico PerLE mi aiuta ad apprendere in modo più efficiente	Items adapted from Sánchez and Hueros (2010)
PUI2	L'ambiente didattico PerLE migliora la mia performance accademica	
PUI3	L'ambiente didattico PerLE rende il mio apprendimento più efficace	
PUI4	L'ambiente didattico PerLE rende il mio apprendimento più semplice	
PUI5	L'ambiente didattico PerLE mi permette di avere più controllo sul mio apprendimento	
PUI6	Complessivamente, l'ambiente didattico PerLE è vantaggioso per il mio apprendimento	
PerLE Ease of Use (PEU)		
PEU1	Imparare ad utilizzare l'ambiente didattico PerLE è semplice per me	

PEU2	È facile reperire i contenuti educativi presenti nell’ambiente didattico PerLE	Items adapted from Abdullah et al. (2016) and Porter and Donthu (2006)
PEU3	Le modalità di utilizzo dell’ambiente didattico PerLe sono chiare e comprensibili	
PEU4	Complessivamente, credo che utilizzare l’ambiente didattico PerLE sia semplice	
PerLE System Usage (PSU)		
PSU1	Uso moltissimo l’ambiente didattico PerLE	Items adapted from Chu and Chen (2016)
PSU2	Sono motivato ad utilizzare l’ambiente didattico PerLE	
PSU3	Molte delle ore della mia giornata le trascorro utilizzando l’ambiente didattico PerLE	
PSU4	Utilizzare l’ambiente didattico PerLE è un’esperienza gratificante	
