# Supplementary material for: PerLE: An “Open Source”, ELearning Moodle-Based, Platform. A Study of University Undergraduates’ Acceptance
Source: Behav Sci (Basel). 2018 Jul 16;8(7):63. doi: 10.3390/bs8070063 (PMC6070931; doi:10.3390/bs8070063)
Supplement: Supplementary file 1 [file behavsci-08-00063-s001.zip › Table S1.pdf]

PerLE: an open source eLearning Moodle-based platform. A study of university undergraduates' acceptance

Supplementary data

Reliability of the measured model S1

Construct	Item	Estimate	S.E.	*p*	R^2	Alpha	CR	AVE
Technical Support (TS)						0.81	0.82	0.53
	TS1	0.73	0.04	<.001	0.54			
	TS2	0.69	0.05	<.001	0.48			
	TS3	0.71	0.04	<.001	0.50			
	TS4	0.76	0.04	<.001	0.58			
PerLE User Interface (PUI						0.90	0.90	0.61
	PUI5	0.71	0.04	<.001	0.50			
	PUI6	0.73	0.04	<.001	0.53			
	PUI7	0.81	0.03	<.001	0.65			
	PUI8	0.82	0.03	<.001	0.67			
	PUI9	0.80	0.03	<.001	0.64			
	PUI10	0.80	0.03	<.001	0.65			
Online Course Lesson (OCL)						0.89	0.88	0.60
	OCL11	0.76	0.04	<.001	0.58			
	OCL12	0.71	0.05	<.001	0.50			
	OCL13	0.73	0.05	<.001	0.53			
	OCL14	0.80	0.03	<.001	0.64			
	OCL15	0.86	0.02	<.001	0.74			
PerLE Usefulness (PU)						0.95	0.95	0.76
	PU16	0.84	0.03	<.001	0.70			
	PU17	0.84	0.02	<.001	0.71			
	PU18	0.92	0.01	<.001	0.84			
	PU19	0.90	0.02	<.001	0.80			
	PU20	0.88	0.02	<.001	0.78			
	PU21	0.86	0.02	<.001	0.74			
PerLE ease of use (PEU)						0.91	0.91	0.71
	PEU22	0.80	0.03	<.001	0.65			
	PEU23	0.85	0.03	<.001	0.72			
	PEU24	0.87	0.02	<.001	0.75			
	PEU25	0.85	0.03	<.001	0.73			
PerLE System Usage (PSU)						0.89	0.90	0.70
	PSU26	0.86	0.03	<.001	0.75			
	PSU27	0.88	0.02	<.001	0.78			
	PSU28	0.73	0.03	<.001	0.53			
	PSU29	0.87	0.02	<.001	0.76			

Note. CR = Composite reliability and AVE = Average Variance Extracted.
